# Solitary schwannoma of the sural nerve: An unusual clinical presentation

**DOI:** 10.3892/etm.2013.1395

**Published:** 2013-11-08

**Authors:** KOSUKE YAMAMOTO, JUN NISHIO, SHINTARO YANO, MASATOSHI NAITO

**Affiliations:** Department of Orthopaedic Surgery, Faculty of Medicine, Fukuoka University, Fukuoka 814-0180, Japan

**Keywords:** enucleation, magnetic resonance imaging, schwannoma, sural nerve

## Abstract

Schwannomas may arise from any peripheral nerve containing Schwann cells. However, sural nerve schwannoma is extremely rare. In this study, a case of solitary schwannoma originating from the sural nerve in a 42-year-old male is presented. Physical examination revealed a 3-cm, elastic-hard, mobile, non-tender mass, while neurovascular examinations, including Tinel’s sign, were normal. Magnetic resonance imaging revealed an oval-shaped subcutaneous mass with iso-signal intensity relative to skeletal muscle on T1-weighted sequences. T2-weighted spectral presaturation with inversion recovery sequences showed higher signal intensity peripherally and lower signal intensity centrally, representing a target sign. Contrast-enhanced T1-weighted sequences demonstrated a marked central enhancement of the mass. The tumor was completely enucleated using an intracapsular technique. Histological examination confirmed the diagnosis of a schwannoma, consisting mainly of Antoni A tissue. The patient had no evidence of local recurrence and no neurological deficit at the final follow-up. Although rare, schwannoma should be considered in the differential diagnosis of a well-defined, oval, subcutaneous mass in the posterior aspect of the lower leg.

## Introduction

Schwannomas are benign peripheral nerve sheath tumors composed exclusively of Schwann cells. The peak incidence occurs in the fourth to the sixth decades of life, with no gender predilection. Patients usually present with a slowly growing mass in the head, neck and the flexor surfaces of the upper and lower extremities (1). Pain and neurological symptoms are uncommon unless the tumor reaches a certain size. The etiology of sporadic schwannoma is not well understood.

The histological hallmark of schwannoma is the alternating pattern of Antoni A and B areas. The relative levels of these two components vary, and they may blend imperceptibly or change abruptly. Antoni A areas are highly cellular and demonstrate nuclear palisading and Verocay bodies. Antoni B areas are less cellular and far less orderly. A number of schwannomas have thick-walled vessels with fibrinoid and hyaline changes in the vessel walls (1).

Sural nerve schwannoma is exceptionally rare. This study presents an unusual case of solitary schwannoma originating from the sural nerve in a middle-aged man. Written informed consent for publication was obtained from the patient.

## Case report

**Figure 1:**
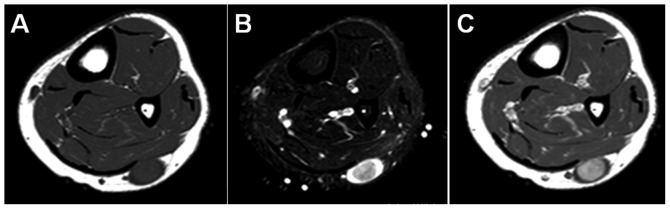
On axial magnetic resonance imaging, the mass had (A) iso-signal intensity relative to adjacent muscle on the T1-weighted sequence and (B) higher signal intensity peripherally and lower signal intensity centrally, representing a target sign, on the T2-weighted spectral presaturation with inversion recovery sequence. (C) Contrast-enhanced T1-weighted sequence showed marked central enhancement of the mass.

A 42-year-old male patient was referred to Fukuoka University Hospital (Fukuoka, Japan) with a >30-year history of a slowly growing, painless mass in the posterolateral aspect of the left distal lower leg. Physical examination revealed a 3-cm, elastic-hard, mobile, non-tender mass, while neurovascular examinations, including Tinel’s sign, were normal. The patient’s past medical history included nothing of note. Magnetic resonance imaging (MRI) demonstrated an oval-shaped subcutaneous mass. The mass showed iso-signal intensity relative to adjacent muscle on T1-weighted sequences (Fig. 1A), and higher signal intensity peripherally and lower signal intensity centrally, representing a target sign, on T2-weighted spectral presaturation with inversion recovery sequences (Fig. 1B). Contrast-enhanced T1-weighted sequences demonstrated a marked central enhancement of the mass (Fig. 1C). Based on these results, a benign neurogenic tumor, such as schwannoma, was strongly suspected.

**Figure 2:**
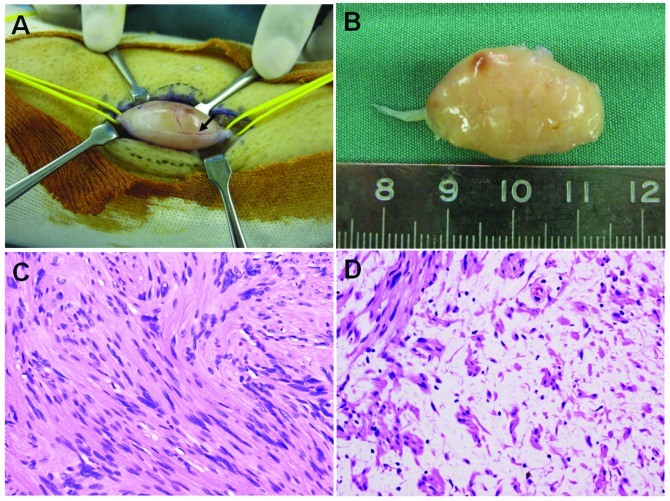
(A) Intraoperative photograph of the tumor and the sural nerve (arrow). (B) Gross appearance of the glistening tan-white tumor. Photomicrographs show (C) hypercellular Antoni A areas and (D) loose Antoni B areas (Hematoxylin and eosin staining; magnification, ×100).

The surgery was performed under general anesthesia with tourniquet control and loupe magnification. The tumor and the proximal and distal portions of the affected nerve were exposed (Fig. 2A), and then a longitudinal incision was carefully made in the epineurium, distal to the fascicles. The epineurial layers were gently peeled out until the shiny surface of the tumor was exposed. The entire tumor mass was subsequently shelled out in one piece without damage to the fascicles. The intraoperative observations were consistent with the diagnosis of schwannoma. Grossly, the smooth-surfaced tumor was tan-white (Fig. 2B). Microscopically, the tumor showed a proliferation of spindle-shaped cells arranged in interlacing fascicles in Antoni A areas (Fig. 2C). Edematous, hypocellular areas, known as Antoni B, were also observed (Fig. 2D). Neither nuclear atypia nor mitotic figures were observed. These features confirmed the diagnosis of schwannoma.

The postoperative course was uneventful. At two months of follow-up, the patient had no evidence of recurrence and no neurological deficit.

## Discussion

The sural nerve is a sensory nerve that lies close to the small saphenous vein and provides sensory innervation to the lateral surface of the foot and ankle. It is typically composed of two merging components: A medial component from the tibial nerve and a lateral component from the lateral sural cutaneous nerve or common peroneal nerve (2). Solitary schwannoma originating from this nerve is extremely rare; only few cases have been described in the English literature (3–5). Moreover, Ogose *et al*(6) described a case of multiple schwannomas in the sural nerve. Despite its rare occurrence, it is important to be aware of the possible existence of sural nerve schwannoma in the lower leg.

The radiographic features of schwannomas are non-specific. On MRI, the lesions usually demonstrate iso- or low signal intensity relative to skeletal muscle on T1-weighted images and high signal intensity on T2-weighted images. A fascicular appearance and a thin hyperintense rim may be observed on T2-weighted images (7). Furthermore, the target sign is one of the characteristic imaging features in schwannomas (8), as observed in the present case. Contrast-enhanced T1-weighted images show strong central enhancement. Previous studies have described a substantial variability in the ^18^F-fluorodeoxyglucose (FDG) uptake by schwannomas (9–11). These studies suggested that schwannomas may not be reliably discriminated from malignant peripheral nerve sheath tumors by FDG positron emission tomography imaging.

In the present case, the possible differential diagnosis included neurofibroma and benign perivascular tumors, such as angioleiomyoma. Neurofibromas may assume one of three growth patterns: Solitary, diffuse or plexiform. Solitary neurofibromas usually arise in a cutaneous nerve of the dermis or subcutis. In the majority of patients, solitary neurofibromas present as a slowly growing, painless mass. Histologically, solitary neurofibromas consist of a mixture of Schwann cells, perineurial cells and fibroblasts in a matrix of wavy collagenous fibers. In contrast to schwannomas, the cells are loosely arranged and diffusely infiltrate the involved nerve (12). On MRI, the lesions usually exhibit iso- or low signal intensity relative to skeletal muscle on T1-weighted images and heterogeneous high signal intensity on T2-weighted images. Contrast-enhanced T1-weighted images show central enhancement. The target sign has been described as being nearly pathognomonic of neurofibroma on T2-weighted images (7,13). In the opinion and experience of the authors, it is often difficult or impossible to distinguish schwannomas from neurofibromas solely on the basis of MRI.

Angioleiomyomas are relatively common soft-tissue tumors that occur in the subcutaneous tissues of the extremities, particularly the lower leg (14). Pain is the major complaint in approximately half of patients. Angioleiomyoma typically presents as a small, slowly growing, firm mass. Histologically, angioleiomyomas are composed of well-differentiated smooth muscle cells with intervening vascular channels. On MRI, the lesions usually exhibit iso- or slightly high signal intensity relative to skeletal muscle on T1-weighted images and heterogeneous high signal intensity on T2-weighted images. Contrast-enhanced T1-weighted images show marked enhancement (15). Unlike neurogenic tumors, angioleiomyomas usually do not exhibit the target sign on T2-weighted images.

In conclusion, a rare example of solitary schwannoma originating from the sural nerve in a middle-aged male patient has been described. Clinicians should consider schwannoma as a possible diagnosis for a well-defined, oval, subcutaneous mass in the posterior aspect of the lower leg.
